# Use of carbon coating on LiNi_0.8_Co_0.1_Mn_0.1_O_2_ cathode material for enhanced performances of lithium-ion batteries

**DOI:** 10.1038/s41598-020-67818-5

**Published:** 2020-07-06

**Authors:** Seong-Ju Sim, Seung-Hwan Lee, Bong-Soo Jin, Hyun-Soo Kim

**Affiliations:** 1 Next Generation Battery Research Center, Korea Electrotechnology Research Institute (KERI), Changwon, Republic of Korea; 2 Department of Advanced Materials Engineering, Daejeon University, Daejeon, 34520 Republic of Korea

**Keywords:** Energy science and technology, Materials science

## Abstract

Ni-rich cathode is one of the promising candidate for high-energy lithium-ion batteries. In this work, we prepare the different super-P carbon black amounts [0.1 (SPB 0.1 wt%), 0.3 (SPB 0.3 wt%), 0.5 (SPB 0.5 wt%) and 0.7 wt% (SPB 0.7 wt%)] of carbon coated LiNi_0.8_Co_0.1_Mn_0.1_O_2_ (NCM811) cathodes and their electrochemical performances are investigated. Carbon coating does not change the crystal structure and morphology of NCM811. Among the coated NCM811, the SPB 0.5 wt% NCM811 delivers the excellent cyclability (87.8% after 80 cycles) and rate capability (86.5% at 2 C) compared to those of pristine NCM811. It is ascribed to that the carbon coating not only increase the Li ion and electron transfer as well as protect the NCM811 cathode materials from side reaction at the electrolyte/NCM811 interface. Therefore, we can conclude that the appropriate amount of carbon coating can be regarded as an effective approach for Ni-rich NCM cathode.

## Introduction

The demand of lithium-ion batteries (LIBs) has been intensively increasing with growing large-scale devices such as electric vehicles (EVs) and energy storage systems (ESSs). Moreover, the high-energy density and long-life LIBs are required for market expansion. To meet the requirements, various cathodes have been studied to increase the energy density of LIBs. Among the cathode materials, Ni-rich NCM cathodes have been considerably researched for high-energy LIBs due to higher specific capacity, relatively low cost and environmental factor compared to other cathode materials^1^. However, Ni-rich NCM cathodes (Ni ≥ 80%) suffer from some problems such as poor cycle life and capacity fading, which limit their commercialization. It is associated with cation mixing, side reactions and residual lithium compounds such as LiOH and Li_2_CO_3_ on the surface of Ni-rich cathodes^2–6^.

To address these problems, many researchers have attempted cation/anion doping for composition modification, surface coating, core–shell and concentration-gradient structures. Among them, the surface-modification with conductive materials such as metal oxides (Al_2_O_3_^7^, ZrO_2_^8^, TiO_2_^9^, SiO_2_^10^), metal phosphate (AlPO_4_^11^, Li_3_PO_4_^12^), fluorides (AlF_3_^13^) and carbon^14^ has been proposed. Many studies have demonstrated that carbon layer such as amorphous carbon, graphene, graphene oxide and carbon black can enhance the electronic conductivity and prevent the side reaction, due to acting as a good physical/chemical barrier against electrolyte^15–19^. In general, a thin carbon layer provides not only high electronic conductivity but also protective effect against structural deformation by elution of transition metal ion. Based on these, carbon layer is an excellent candidate to enhance the electrochemical performances. There are several ways to form carbon layer such as mechanical milling, thermal decomposition, chemical vapor deposition (CVD) and pyrolysis of adsorbed organic compounds^20,21^. However, these methods are not suitable for mass production because they need complex process and expensive equipment.

In the present study, carbon coated NCM811 was successfully prepared using carbon black (super-P) as a carbon sources through a simple one-step process and we investigate the effect of carbon coating on the electrochemical performances. The carbon coated NCM811 shows the superior electrochemical performances and these findings indicate that carbon coating is one of the effective way for high performance and stability of Ni-rich NCM cathode.

## Experimental

**Figure 1:**
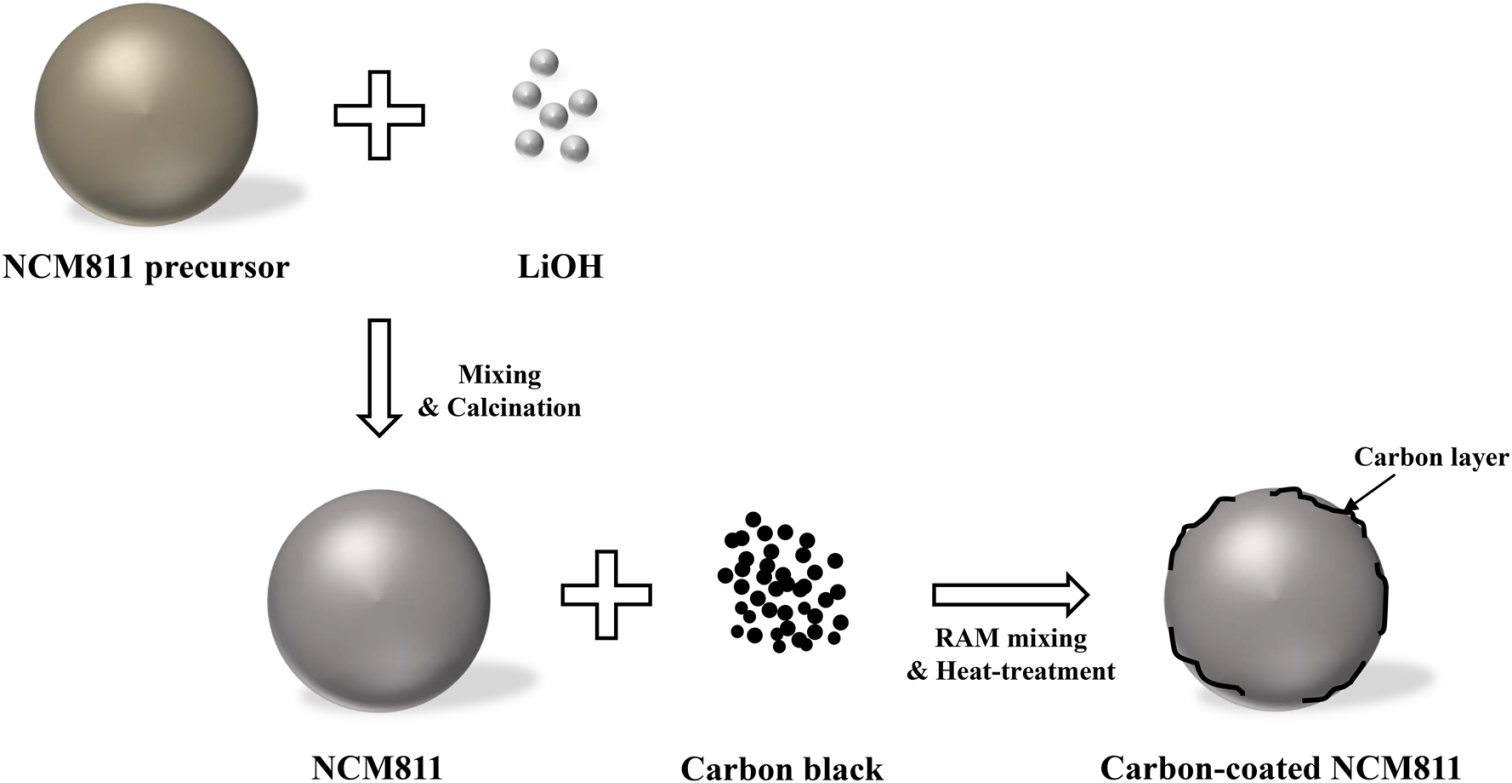
Schematic illustration of the synthesis process of carbon-coated NCM811.

To prepare the NCM811, the Ni_0.8_Co_0.1_Mn_0.1_(OH)_2_ precursor was fabricated via a co-precipitation method. The solutions of NiSO_4_·6H_2_O, CoSO_4_·7H_2_O and MnSO_4_·H_2_O were used as starting materials. The NaOH and NH_4_OH solution were also used as chelating agent. As-prepared spherical Ni_0.8_Co_0.1_Mn_0.1_(OH)_2_ precursor was mixed with LiOH·H_2_O in a molar ratio of 1:1.05. Then, the mixed powders were calcined at 480 °C for 5 h and 750 °C for 15 h in air, as shown in Fig. 1^6^. The powders were mixed with different amounts (SPB 0.1 wt%, SPB 0.3 wt%, SPB 0.5 wt% and SPB 0.7 wt%) of super-P carbon black via resonant acoustic mixer (PharmaRAM™ I, Resodyn Acoustic Mixers Inc.) at the acceleration of high mix for 20 min. and then calcined at 300 °C for 3 h.

The structural properties and morphologies of the cathodes were measured via X-ray diffraction (XRD, X-pert PRO MPD, Philips, Cu Kα), field emission scanning electron microscopy (FESEM, S-4800, HITACHI) and field emission transmission electron microscope (FETEM, Titan G2, FEI Company).

To measure the electrochemical performance, the cathodes were prepared using 96 wt% active materials, 2 wt% super-P and 2 wt% polyvinylidene fluoride (PVDF) binder. The prepared slurry was coated on Al foil (16 μm in thickness) and then dried at 100 °C for 10 h in a vacuum oven. The cathodes were punched into disks and then dried at 120 °C for 10 h. The 2032 coin cells were fabricated by pristine and carbon-coated NCM811 cathode and lithium (500 μm in thickness) anode. A polyethylene (PE, 20 μm in thickness) was employed as a separator and 1 M LiPF_6_ in a mixed solution containing ethylene carbonate (EC)/dimethyl carbonate (DMC)/ethylmethyl carbonate (EMC) (1:1:1, v/v/v) was used as electrolyte. The coin cells were assembled in an Ar-filled glove box^6^.

The charge–discharge performance was galvanostatically tested in the voltage range of 3.0–4.3 V and various current density using electrochemical equipment (TOSCAT-3100, Toyo system) at room temperature. Cyclic voltammetry (CV) of the samples was performed with multi potentiostat (VSP300, Bio-Logic) at a scan rate of 0.1 mV s^−1^. The electrochemical impedance spectroscopy (EIS) measurement was tested with a VSP300 impedance analyzer in the frequency range of 1 MHz–10 mHz with 5 mV amplitude^6^.

## Results and discussion

**Figure 2:**
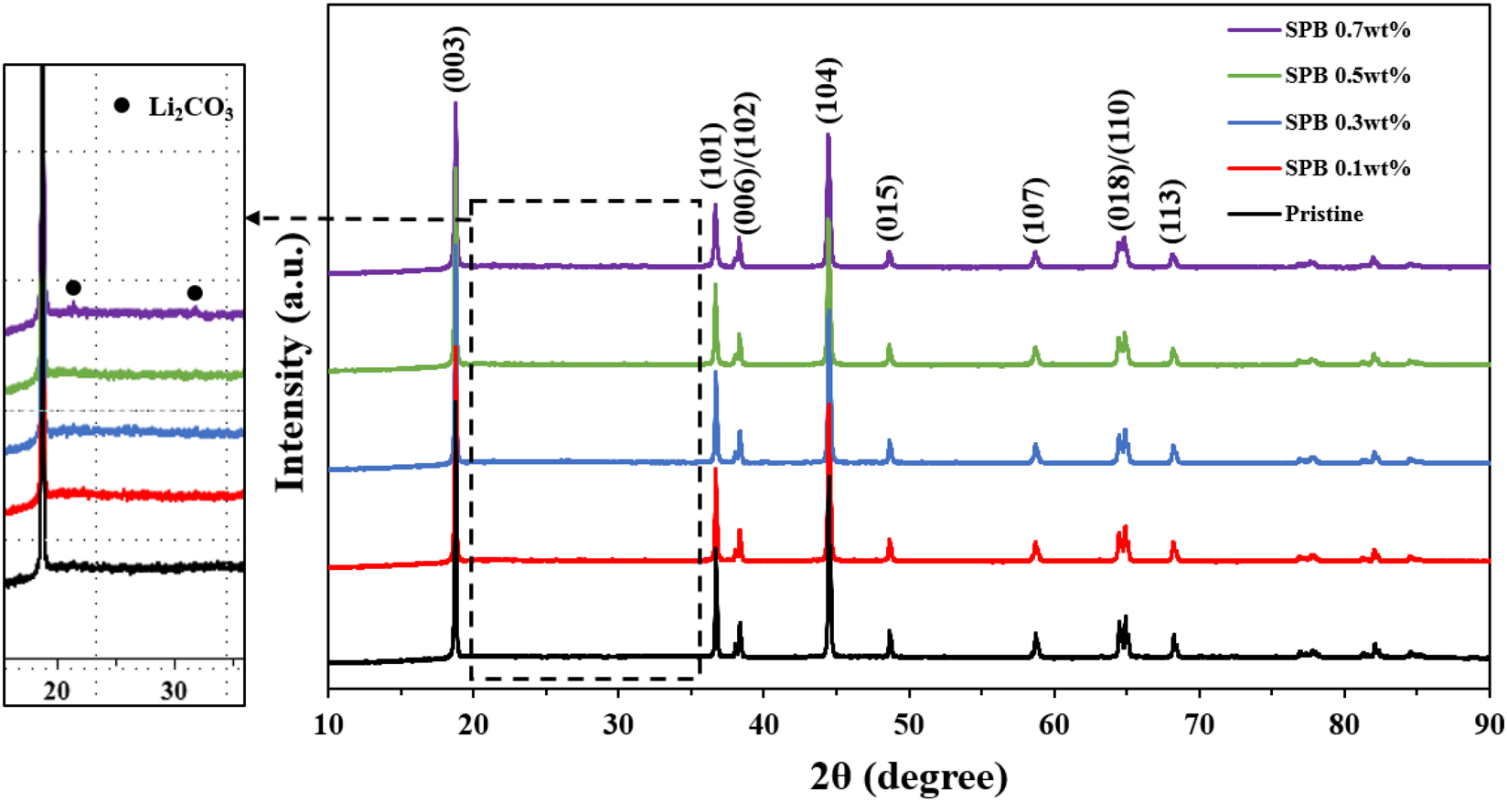
XRD patterns of pristine and carbon-coated NCM811.

Figure 2 shows the XRD patterns of pristine and carbon-coated NCM811. All peaks are indexed based on a layered hexagonal α-NaFeO_2_ structure with the space group R${\overline{\text{3}}}$m^22^. The clear splitting of (006)/(102) and (018)/(110) peaks is observed in all samples, indicating well-ordered layered structure with small cation mixing^23^. However, the impurity phase Li_2_CO_3_, which is responsible for the deterioration of electrochemical properties, is observed for SPB 0.7 wt% NCM811. It can be explained by the large amount carbon sources can produce Li_2_CO_3_ by reacting with Li_2_O (lithium residuals), as the following equation^24^:

1 $${\text{C }} + {\text{ O}}_{2} + {\text{ Li}}_{2} {\text{O }} \to {\text{ Li}}_{2} {\text{CO}}_{3} .$$

This phenomenon leads to excessive lithium impurities on the surface of NCM811 and is closely related to the crystallinity of the layered NCM811 structure. The intensity ratio of I(003)/I(104) means the degree of cation mixing between Li^+^ and Ni^2+^ in the Li layer due to the similar ionic radius of Li^+^ (0.76 Å) and Ni^2+^ (0.69 Å). A higher value the I(003)/I(104), the lower cation mixing. It was reported that I(003)/I(104) value less than 1.2 indicates undesirable cation mixing, resulting in poor electrochemical performances^25,26^. As summarized in Table 1, the I(003)/I(104) values are inversely proportional to the carbon content and SPB 0.7 wt% NCM811 shows the lowest value of 1.35. However, all samples deliver high I(003)/I(104) values, indicating excellent layered structures with high cation ordering. Therefore, it is clear that appropriate amount of carbon coating does not adversely affect the structure of NCM811.

**Table 1:** The I_003_/I_104_ ratio of pristine and carbon-coated NCM811 samples.

	Pristine	0.1 wt%	0.3 wt%	0.5 wt%	0.7 wt%
I_003_/I_104_	1.43	1.42	1.42	1.41	1.35

**Figure 3:**
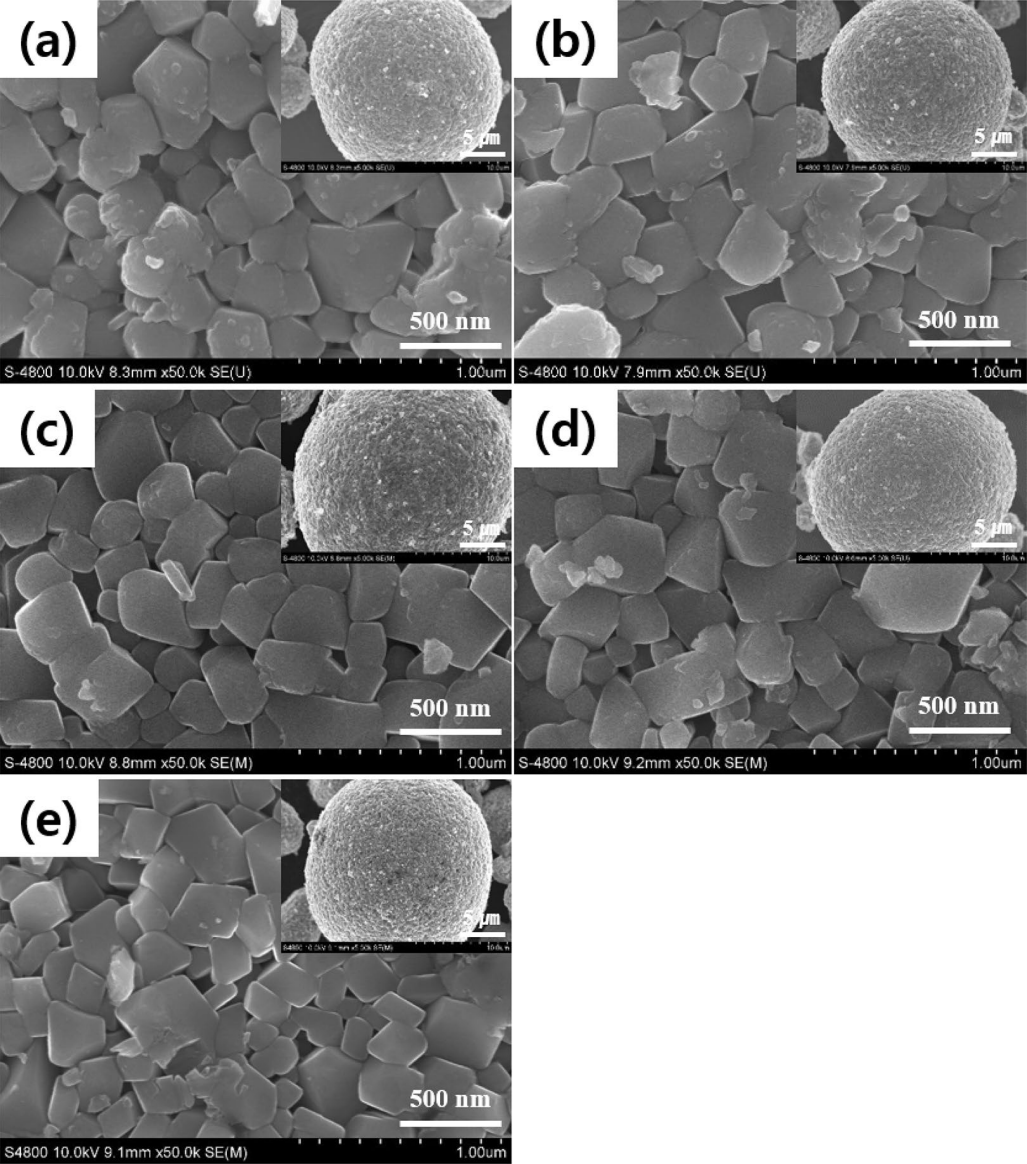
FESEM images of pristine and carbon-coated NCM811: (**a**) pristine; (**b**) SPB 0.1 wt%; (**c**) SPB 0.3 wt%; (**d**) SPB 0.5 wt% and (**e**) SPB 0.7 wt%.

The microstructures of the pristine and carbon-coated NCM811 are shown in Fig. 3. The images show the micro-sized spherical secondary particles (15–20 µm), aggregated with primary particles of 200–500 nm. It is the typical shape of cathode powders by the co-precipitation method. There are no clear differences in the morphologies between the pristine and carbon-coated NCM811. The size of primary particles of all samples is almost similar regardless of carbon contents. Therefore, it can be inferred that the carbon coating does not affect the grain growth. The secondary particle with a porous structure has high specific surface areas and pore volumes between primary particles, resulting in improving the electrochemical performances by excellent electrolyte wettability of NCM811.

**Figure 4:**
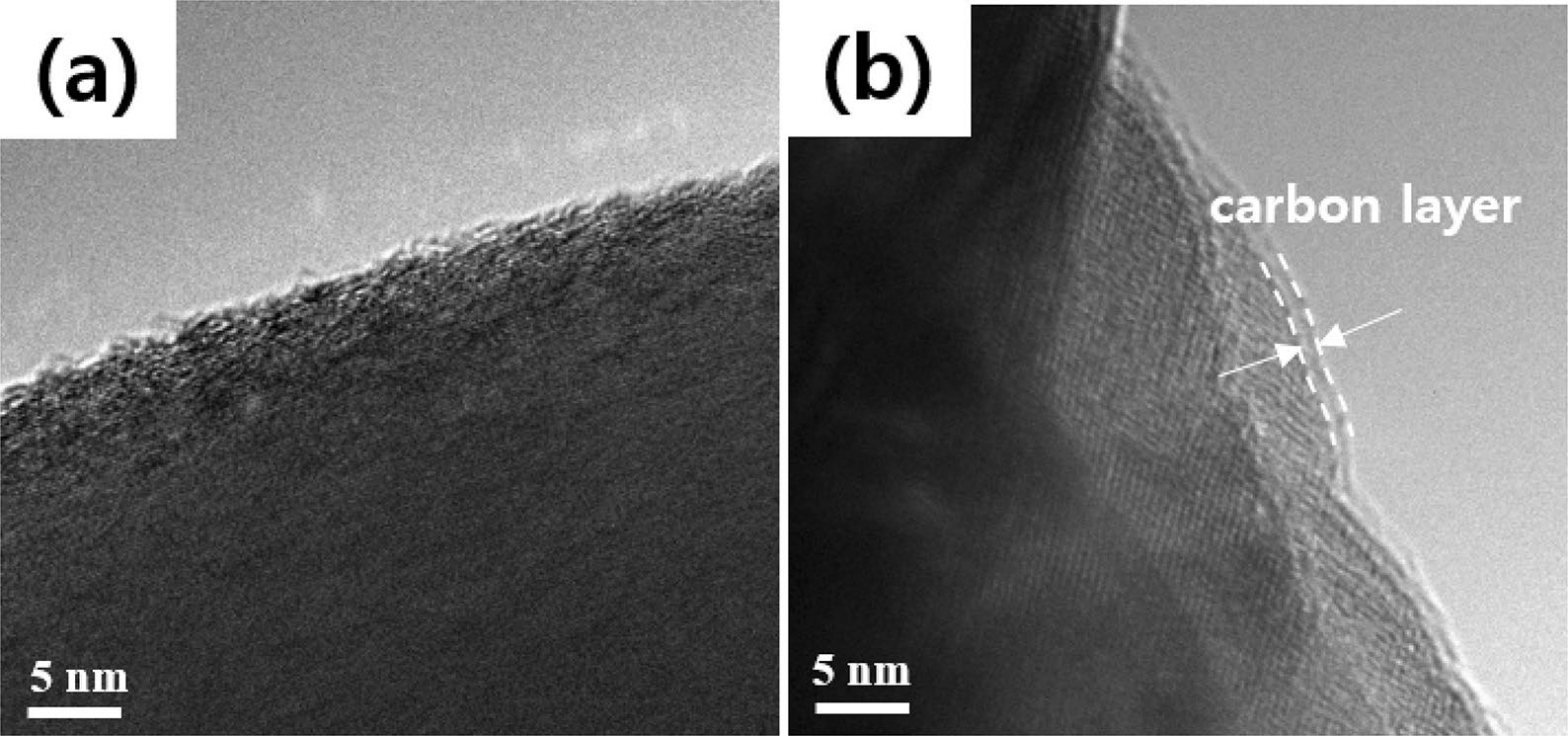
FETEM images of (**a**) pristine and (**b**) SPB 0.5 wt% NCM811.

Figure 4 shows the FETEM images of the (a) pristine and (b) SPB 0.5 wt% NCM811 to identify the coating layers on the surface of NCM811. The pristine NCM811 shows the perfect crystallinity without amorphous layer on the surface of the NCM811, indicating no carbon layer. Comparatively, it is clear that SPB 0.5 wt% NCM811 exhibits amorphous carbon coating on the surface. The NCM811 was randomly coated with a carbon layer ranging from 0.89 to 1.23 nm. Hong et al. reported that it is not easy to form the uniform and ultrathin carbon layer due to weak adhesion^27^.

The electrochemical performances of the NCM811 cathode were measured in thick electrode laminates with high mass loading per area (approximately 15.1 mg/cm^2^) because the high areal capacity is the one of the most important factor for practical application. Also, the total weights of carbon-coated NCM811 include a carbon weight.

**Figure 5:**
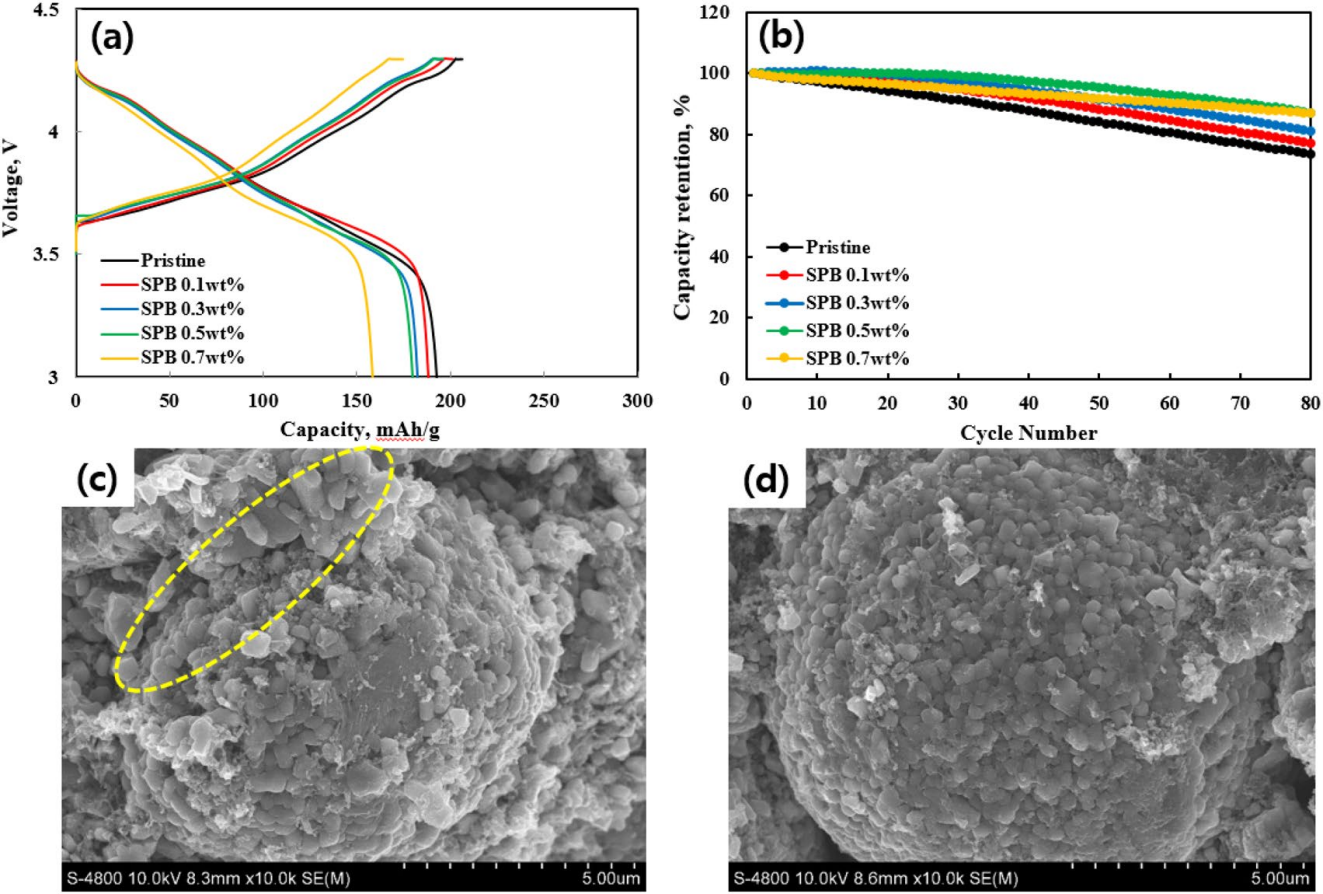
(**a**) Initial charge–discharge curves at 0.5 C and (**b**) cycle performance of pristine and carbon-coated NCM811. FESEM images of (**c**) pristine and (**d**) SPB 0.5 wt% NCM811 after 80 cycles.

Figure 5 shows the (a) initial charge–discharge profiles and (b) cycling performances of pristine and carbon-coated NCM811 samples at the rate of 0.5 C (1 C = 202 mAh g^−1^) at 25 °C. The voltage plateaus correspond to the typical charge–discharge behavior of the Ni-rich layered NCM cathodes. The pristine NCM811 initially delivered a discharge capacity of 192.8 mAh g^−1^, while the discharge capacities of carbon-coated NCM811 slightly decrease. The SPB 0.1, 0.3, 0.5 and 0.7 wt% NCM811 exhibited the initial discharge capacities of 190.5, 189.1, 188.6 and 165.2 mAh g^−1^, respectively. This is because carbon coating layer serves as an obstacle because of higher electrode polarizations. It leads to inferior specific capacities compared to pristine NCM811. Among them, the capacity of SPB 0.7 wt% NCM811 drastically decreased due to holdback for the lithium ion transport by excessive thickness of the carbon layer.

Figure 5b shows the cycle performances of pristine and carbon-coated NCM811 samples after 80 cycles at 0.5 C. Obviously, the carbon coating helps to obtain a superior cycle stability of NCM811 compared to pristine NCM811. The capacity retentions of SPB 0.1, 0.3, 0.5 wt% NCM811 were 77.8, 81.9 and 87.8% after 80 cycles. The capacity retentions of SPB 0.1 and 0.3 wt% NCM811 were somewhat lower than those of SPB 0.5 wt% and 0.7 wt% NCM811. It can be explained by insufficient coating coverage on the NCM811 surface^28^. In addition, although the SPB 0.7 wt% NCM811 also exhibited excellent cyclability, it should be mentioned that the discharge capacity was too low compared to other samples, as mentioned above. Most importantly, it is clearly showed that the carbon-coated NCM811 shows stable cyclability than that of pristine NCM811. The pristine NCM811 had a capacity retention of 74.3% under the same condition. Among the carbon-coated NCM811, the SPB 0.5 wt% NCM811 can minimize the capacity fading due to the fast diffusion kinetics of lithium ions and electrons. Also, it can be attributed to the carbon coating, which suppresses the side reaction between NCM811 and electrolyte, resulting in structural degradation. More importantly, it is closely related to the gas generation, resulting from lattice oxygen, Li_2_CO_3_ and electrolyte decomposition^29^. (1) CO and CO_2_ are anodic oxidation product of EC and DMC solvent, which can be expressed as^29^:

2 $${\text{O}}_{{{\text{lattice}}}} + {\text{ EC/DMC}} \to {\text{2CO}}_{{2}} + {\text{ CO }} + {\text{2H}}_{{2}} {\text{O}}$$

3 $${\text{2O}}_{{{\text{lattice}}}} \to {\text{O}}_{{2}}$$

(2) It is inevitable that Li_2_CO_3_ is formed which reacts with LiPF_6_ to generate POF_3_ and CO_2_ as shown in the following equation^29^:

4 $${\text{LiPF}}_{{6}} + {\text{ Li}}_{{2}} {\text{CO}}_{{3}} \to {\text{POF}}_{{3}} + {\text{ CO}}_{{2}} + {\text{ 3LiF}}$$

In addition, the LiPF_6_ salt is spontaneously decomposed and generates gas as follows^29^:

5 $${\text{LiPF}}_{{6}} \to {\text{LiF}} + {\text{PF}}_{{5}}$$

6 $${\text{PF}}_{{5}} + {\text{H}}_{{2}} {\text{O}} \to {\text{POF}}_{{3}} + {\text{2HF}}$$

7 $${\text{2POF}}_{{3}} + {\text{ 3Li}}_{{2}} {\text{O}}^{ - } \to {\text{6LiF }} + {\text{P}}_{{2}} {\text{O}}_{{5}} \left( {{\text{or Li}}_{{\text{x}}} {\text{POF}}_{{\text{y}}} } \right)$$

Most importantly, carbon layer is expected to protect the NCM811 from attack by hydrogen fluoride (HF), dissolving the transition metal ions, thereby collapses the NCM811 structure. Therefore, carbon coating can effectively suppress the capacity decay and solve the safety problems (explosion and fires)^30^.

Figure 5c,d show the FESEM images of the pristine and SPB 0.5 wt% NCM811 after 80 cycles, indicating the effect of carbon coating for electrode stability. After cycling, the pristine and SPB 0.5 wt% NCM811 exhibited a clear difference. The pristine NCM811 showed separation of the primary particles from the secondary particles (marked by yellow circle). It is well known that Ni-rich layered NCM cathodes, especially x > 0.8, suffer from volume change during charge/discharge process, which creates external surface cracks and separation of primary particles. The external surface cracks gradually deteriorate the capacity by electrolyte attack and internal cracks^31^. However, the SPB 0.5 wt% NCM811 maintained its original shape with no significant damage, enabling superior electrochemical performances.

**Figure 6:**
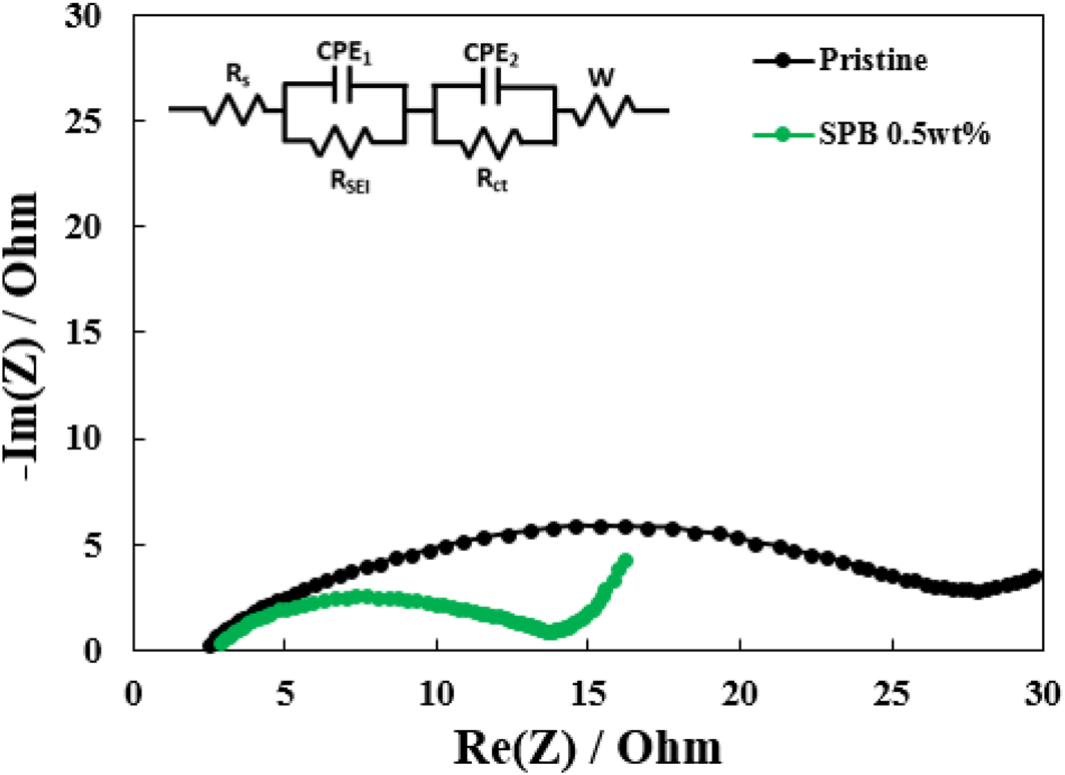
Nyquist plots of pristine and SPB 0.5 wt% NCM811 samples after 50 cycles.

To further investigate the effect of the carbon coating on the NCM811/electrolyte interface resistance, the electrochemical impedance spectroscopy (EIS) was performed after 50 cycles (Fig. 6). The semicircle at the highest frequency is related to the resistance of solid electrolyte interface (R_SEI_), the high-to-medium frequency semicircle represent the charge transfer resistance at the interface between electrode and electrolyte (R_ct_), and a slope at low frequency corresponds to the Warburg impedance, related to the Li^+^ diffusion in the solid electrode^30^. According to the literature^32^, the impedance of the cell was mainly decided by the cathode impedance, especially for R_ct_. Moreover, the SPB 0.5 wt% NCM811 shows the lower R_ct_ values than pristine NCM811, as shown in Table 2. It can be inferred that carbon layer can reduce the side reaction and maintain the original structure of well-ordered NCM811 structure, resulting in smooth and rapid lithium ion and electron transfer.

**Table 2:** The R_ct_ values of pristine and carbon-coated NCM811 samples after 50 cycles.

	R_ct_ [Ω]
	After cycling
Pristine	23.65
SPB 0.5 wt%	11.28

**Figure 7:**
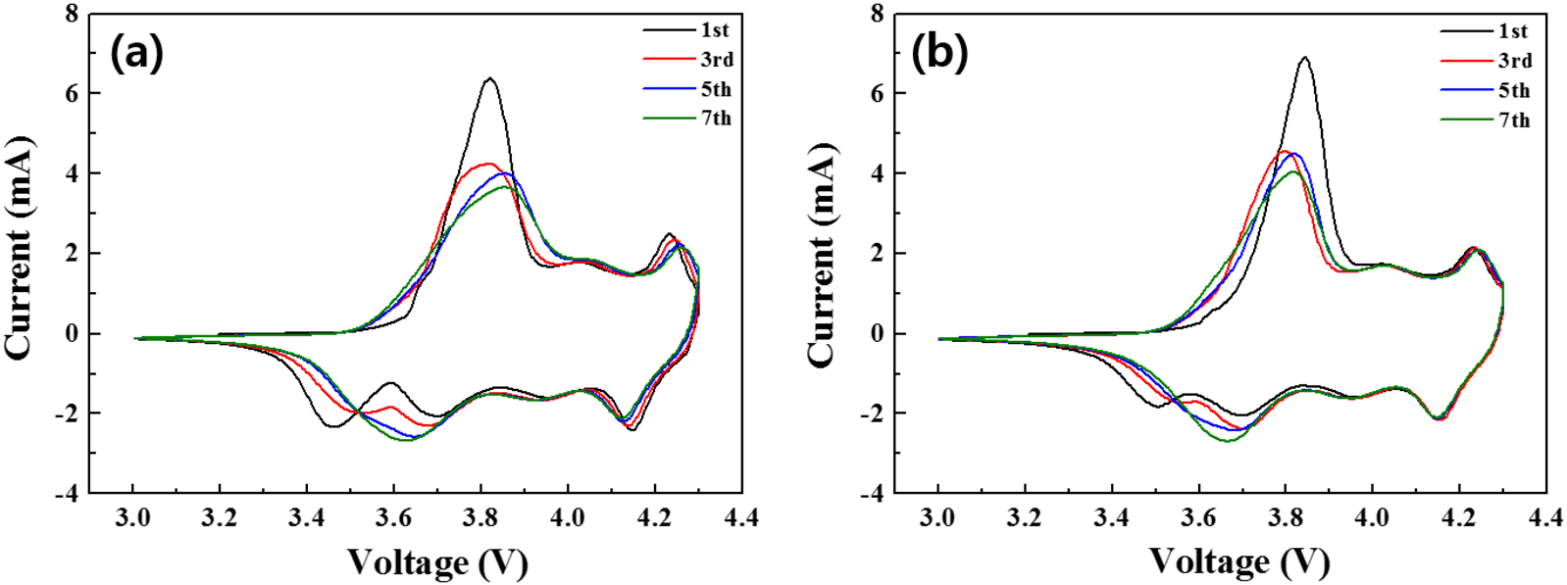
Cyclic voltammetry of (**a**) pristine and (**b**) SPB 0.5 wt% NCM811 in the voltage range of 3.0–4.3 V at a scan rate of 0.1 mV s^−1^.

Figure 7a,b show the CV curves of pristine and SPB 0.5 wt% NCM811 samples after 1, 3, 5 and 7 cycles at a scan rate of 0.1 mV s^−1^. During the charge–discharge process, the pristine and SPB 0.5 wt% NCM811 had oxidation/reduction peaks at around 3.8 V and 4.23 V, which are corresponding to Ni^2+^/Ni^4+^ and Co^3+^/Co^4+^, respectively^33,34^. It can be seen that the position of the redox peak of the carbon coating is more stable than that of the pristine NCM811^35^. It demonstrates that carbon coating provides lower electrode polarization between anodic and cathodic peaks, enabling better reversibility during cycling^36^. These low R_ct_ and polarization values of SPB 0.5 wt% NCM811 are the important reasons for superior capacity retention during long-term cycling.

**Figure 8:**
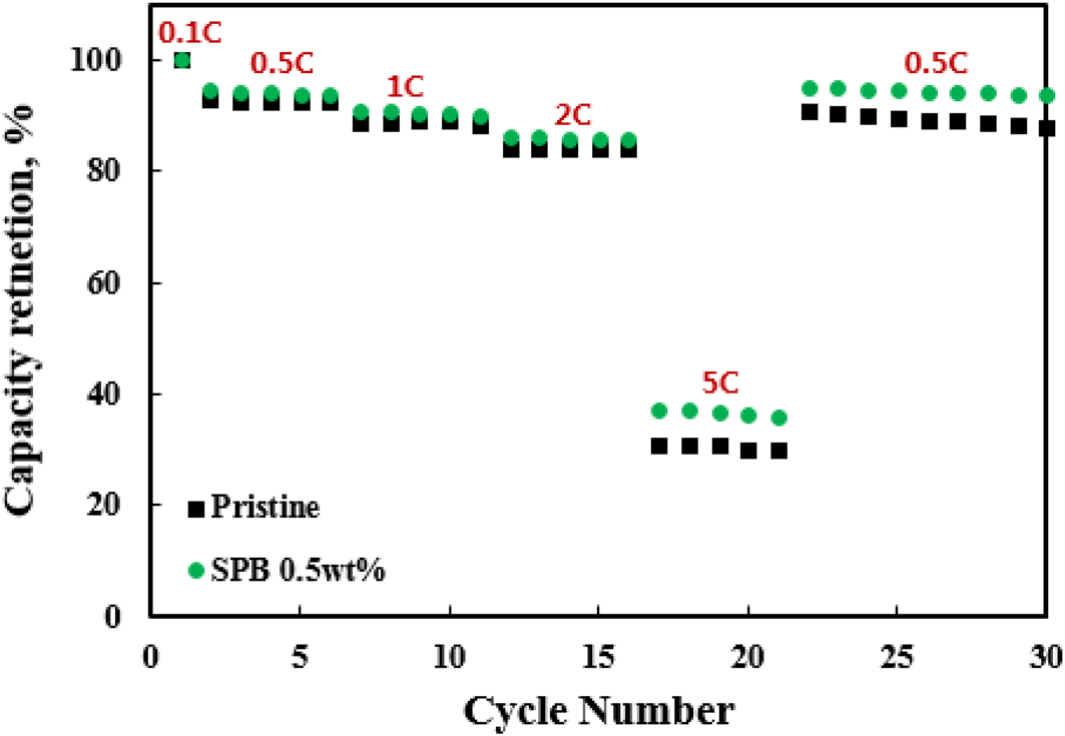
Rate capability of the pristine and SPB 0.5 wt% NCM811.

Figure 8 shows the rate capability of the pristine and SPB 0.5 wt% NCM811. It is obvious that the retention decreases with the increasing C-rates. All samples showed comparable retentions up to 2 C. However, a significant difference is shown between the two samples at 5 C. The SPB 0.5 wt% NCM811 showed relatively higher retention (37.8 %) while the pristine NCM811 maintained lower retention of 30.9%. The superior capacity retention is attributed to the higher conductivity of SPB 0.5 wt% NCM811. Moreover, SPB 0.5 wt% NCM811 entirely recovered the capacity retention when the current density returned to 0.5 C, resulting from modification of NCM811 surface chemistry. It can be explained by the fact that a carbon coating layer of appropriate thickness inhibits negative effects, thereby improving structural stability and decreasing resistance. These enable quick lithium ion and electron migration^29^. Therefore, we can conclude that the carbon coating can improve the rate capability and reversibility, especially at the high rate. It is one of the most important factor for application to the high-power LIBs.

## Conclusion

In this paper, we prepared pristine and carbon-coated NCM811 cathodes. The appropriate thickness of carbon layer effectively maintained the electrode stability and delivered better cyclability and rate capability than pristine NCM811. Among the carbon-coated NCM811, the SPB 0.5 wt% NCM811 had not only the original well-crystallized structure but also superior electrochemical performances. These can be explained by the dual roles of carbon coating, including (1) physical and chemical barrier and (2) increase in Li^+^ and electron conductivity. Based on these, the carbon coating is remarkable breakthrough to overcome the drawbacks of Ni-rich cathode.
